# Socioeconomic inequality for hypertension among reproductive age women aged 15–49 from five Sub-Saharan Africa countries: A decomposition analysis of DHS Data

**DOI:** 10.1371/journal.pgph.0004738

**Published:** 2025-07-02

**Authors:** Daniel Bekele Ketema, Desalegn Markos Shifti, Teketo Kassaw Tegegne, Daniel Bogale Odo, Subash Thapa, Abel Dadi, Tahir Ahmed Hassen, Getiye Dejenu Kibret, Zemen Y. Kassa, Erkihun Amsalu, Yonatan M. Mesfin, Habtamu Mellie Bizuayehu, Sewunet Admasu Belachew, Meless Gebrie Bore, Abdulbasit Seid, Kedir Y. Ahmed

**Affiliations:** 1 College of Medicine and Health science, School of Public Health, Debre Markos University, Debre Markos, Ethiopia; 2 Child Health Research Centre, The University of Queensland, South Brisbane, Australia; 3 Institute for Physical Activity and Nutrition, Deakin University, Geelong, Victoria, Australia; 4 National Centre for Aboriginal and Torres Strait Islander Wellbeing Research, National Centre for Epidemiology and Population Health, Australian National University, Canberra, Australia; 5 Rural Health Research Institute, Charles Sturt University, Orange, New South Wales, Australia; 6 Menzies School of Health research, Charles Darwin University, Brinkin, Australia; 7 Addis Continental Institute of Public Health, Addis Ababa, Ethiopia; 8 College of Health, Medicine and Wellbeing University of Newcastle, Australia; 9 Centre for Health Systems and Safety Research, Australian Institute of Health Innovation, Faculty of Medicine, Health and Human Sciences, Macquarie University, Sydney, Australia; 10 College of Medicine and Health Sciences, Hawassa University, Hawassa, Ethiopia; 11 Sydney Medical School, Faculty of Medicine and Health, University of Sydney, Sydney, Australia; 12 Paul Hospital Millennium Medical College, Addis Ababa, Ethiopia; 13 Immunity, & Global Health, Murdoch Children’s Research Institute (MCRI), Parkville Victoria, Australia; 14 The First Nations Cancer & Wellbeing Research (FNCWR) Program, School of Public Health, The University of Queensland, Brisbane, Australia; 15 School of Nursing and Midwifery, Faculty of Health, University of Technology Sydney, Ultimo, New South Wales, Australia; 16 School of Nursing, College of Medicine and Health Science, Hawassa University, Hawassa, Ethiopia; 17 School of Public Health and Prevention Medicine, Monash University, Melbourne, Victoria, Australia; University College London, UNITED KINGDOM OF GREAT BRITAIN AND NORTHERN IRELAND

## Abstract

Hypertension is a significant public health issue in sub-Saharan Africa, especially among reproductive-aged women. This study aims to assess and decompose socioeconomic inequalities in hypertension across five SSA countries using DHS data. Our study analyzed the latest Demographic and Health Survey data from five SSA countries, including Benin, Cameroon, Ghana, Kenya, and Lesotho. We selected these five SSA countries based on data availability and conducted analyses using the Erreygers normalized concentration index (ECI) and concentration curve to measure and decompose inequalities.A weighted total sample of 52,076 reproductive-age women in SSA were included. The overall weighted ECI was estimated to be 0.06 (95% confidence interval (CI):0.049; 0.065). Key contributors to socioeconomic inequalities in hypertension include wealth quantile (126%), media exposure (19%), and educational attainment (2.3%), marital status (-34.3%), and residence (-31.7).In conclusion, there is a clear pro-rich socioeconomic inequality in hypertension among reproductive-age women in Sub-Saharan Africa. Wealth index, marital status, media exposure, and place of residence are the primary drivers of this disparity. Addressing these socioeconomic disparities through targeted interventions can significantly reduce hypertension rates among reproductive-aged women in SSA.

## Background

Hypertension, often known as high blood pressure, is a growing public health challenge in Sub-Saharan Africa (SSA), with its burden shifting from high-income countries to low- and middle-income countries [1]. In 2020, the prevalence of hypertension among adults aged 25 and older was 46%, surpassing rates in the Americas and other high-income countries [2]. Socio-behavioral factors such as increased sodium intake, reduced physical activity, financial burden, and underlying medical conditions such as obesity are associated with hypertension [3].

Hypertension can lead to serious health consequences including causing cardiovascular complications, such as angina, myocardial infarction, heart failure, stroke, peripheral artery disease, and abdominal aortic aneurysm [4–6]. Hypertension in reproductive-aged women poses severe health risks, including complications during pregnancy and increased mortality [7,8]. Previous studies indicate rising hypertension prevalence in SSA [9–11], but socioeconomic disparities remain under studied.

Previous studies examining the relationship between socioeconomic status and hypertension reported contradictory findings. Studies from both developing as well as developed countries [12–15], reported that higher socioeconomic status is associated with a higher prevalence of hypertension, due to sedentary lifestyles and increased accessibility to high-fat diets. Conversely, others have shown the opposite effect, indicating that lower socioeconomic households are at a higher risk of hypertension [16–18]. While these studies have explored hypertension prevalence, few have systematically assessed socioeconomic inequalities in hypertension among reproductive-aged women in SSA. This study fills this gap by comprehensively analysed the socioeconomic disparities for hypertension. Therefore, this study aims to (1) measure socioeconomic inequalities in hypertension among reproductive-aged women in five SSA countries, and (2) identify key contributors to these inequalities

Understanding patterns of socioeconomic inequalities is essential for developing targeted interventions for addressing the underlying causes of hypertension disparities and promoting equitable health outcomes among reproductive-age women in SSA.

## Methods

### Data and study setting

This study used the latest Demographic and Health Survey (DHS) data from five SSA countries. We selected Ghana, Kenya, Nigeria, Tanzania, and Zambia due to the availability of recent DHS data. However, this selection may limit the generalizability of our findings to other SSA countries. DHS conducted in more than 90 low- and middle-income countries with the main aims of providing demographic and health data for policymaking and national health programs. The high-quality interviewer training and standardized data collection tools across countries contribute to the reliability of the data [19]. The DHS gathers data on the demographics and health of individuals, encompassing topics such as maternal and child health, mortality, nutrition, and the social determinants of health [20].

### Sampling procedures and populations

The DHS uses a two-stage stratified cluster sampling technique to ensure national representativeness. In the first stage, clusters/ Enumeration Areas (EA) were randomly selected from the sampling frame (commonly obtained/from the most recent national census). In the second stage, households in each cluster or EA were selected using systematic random sampling. The detailed sampling procedures are available in the measure DHS guide [21]. Further details about the sampling design and questionnaire can be found in the country-specific Monitoring and Evaluation to Assess and Use Results DHS reports. For this study, a total weighted sample of 52,076 reproductive-age women was included, distributed as follows: Ghana (9,394), Cameroon (13,616), Kenya (16,716), Lesotho (4,645), and Benin (7,706).

### Outcome variables

Hypertension was self-reported based on medical diagnosis, which may introduce reporting bias. We considered women aged 15–49 years for analysis.

### Socioeconomic measure

We used the household wealth index as a comprehensive indicator of the socioeconomic position and living standards of the respondents. In the DHS surveys PCA was used to construct a wealth index based on household assets, categorized into quintiles. This method captures relative socioeconomic status within each country. This wealth index was constructed by assessing using the following household assets such as toilets, electricity, television, radio, fridge, and bicycle, as well as the availability of a source of drinking water and floor material of the main house [22]. The resulting continuous-scale wealth index was subsequently divided into five quintiles: the poorest (1^st^ quintile), poorer (2^nd^ quintile), middle (3^rd^ quintile), richer (4^th^ quintile), and richest (5^th^ quintile). Table 1 below provides a comprehensive overview of the wealth index distribution for each of the included countries.

**Table 1:** Distribution of wealth index among reproductive age women in five SSA countries from their latest DHS.

Countries (DHS Year)	Wealth index n(%)					
	Poorest	Poorer	Middle	Richer	Richest	Total
Benin (2013)	1424 (18.48)	1551 (20.13)	1422 (18.45)	1574 (18.45)	1734 (20.44)	**7,706**
Cameroon (2018)	2239 (16.44)	2502 (18.37)	2696 (19.80)	2938 (21.58)	3241 (23.80)	**13,616**
Ghana (2014)	1512 (16.09)	1636 (17.42)	1937 (20.62)	2117 (22.54)	2192 (23.33)	**9394**
Kenya (2022)	2599 (15.55)	2974 (17.80)	3086 (18.46)	3729 (22.31)	4328 (28.32)	**16,716**
Lesotho (2014)	595 (12.80)	710 (15.29)	883 (19.00)	1141 (24.56)	1316 (28.33)	**4,645**
**Total**	**8369 (16.07)**	**9373 (18.00)**	**10,024 (19.24)**	**11,499 (22.08)**	**12,811 (25.00)**	**52,076**

### Other explanatory variables

Variables included age, education, marital status, and media exposure (frequency of newspaper, radio, and television use). Media exposure was assessed based on DHS questions about the frequency of media consumption.

### Data management and analysis

Data were managed and analyzed using STATA version 18 due to its robust capabilities for complex survey data and advanced statistical techniques.“. The data management and coding procedures strictly adhered to the well-established guidelines outlined by the DHS statistics [22] and existing literature. Descriptive statistics and frequency distributions were used to describe participant characteristics. Reported estimates were weighted by sampling weights to account for the complex survey design of DHS.

### Definition of terms

Absolute contribution: The actual value by which a specific factor (e.g., education, place of residence) contributes to the measured inequality in hypertension. It is calculated by multiplying elasticity to a given determinant with the level of socioeconomic inequality (concentration index) in that determinant

Percent contribution: The proportion of the total explained inequality that is attributed to a specific factor.

Sum contribution: The sum of percent contributions from all explanatory variables. It shows how much of the inequality is explained by the model.

### SEI measurement

SEI was measured using the concentration index and concentration curve [23]. Concentration index (CI) to quantify and compare the degree of socioeconomic inequality of hypertension, which is the most appropriate measure of health inequality [24,25]. The CI measures socioeconomic inequality in health, with values ranging from -1–1. The Erreygers normalized concentration index (ECI) adjusts for the bounded nature of binary health outcomes, providing a more accurate measure of inequality [26,27]. The ECI along with its 95% confidence interval were calculated to assess the statistical significance of the difference between the concentration curve and the line of perfect equality.

Mathematically, ECI can be defined as:


ECI=4*μ*CI(y).

Where ECI is Erreygers concentration index, CI(y) stands for the generalized concentration index and μ is the mean of the health variable, in this case, hypertension. Then, the ECI with its 95% confidence interval was reported in this study. Moreover, the ECI was calculated to determine the relative contribution of numerous factors to socioeconomic inequalities in hypertension [23,28,29]. Hence, for any linear additive regression model of health outcome (y) [29],


$$y=\mu +\sum_{k}\beta_{k}X_{k}+\in$$

The concentration index for y, CI, is computed as:


$$y=\sum_{k}\left(\frac{\beta_{k}{\overset{―}{X}}_{k}}{\mu }\right)C_{k}+\frac{{gc}_{\in }}{\mu }$$

Where “y” denotes the health outcome variable (in this case, SEI in hypertension), $X_{k}$ is a set of the socioeconomic determinants of the health outcome, α is the intercept, $\beta_{k}$ is the coefficient of $X_{k}$, µ is the mean of y, ${\overset{―}{X}}_{k}$ is the mean of $X_{k}$, $C_{k}$ is the CI for $X_{k}$, ${gc}_{\in }$ is the generalized CI for the error term (∈), $\frac{\beta_{k}{\overset{―}{X}}_{k}}{\mu }$ is the elasticity of y concerning ${\overset{―}{X}}_{k}$ [28,30].

The concentration curve represents the cumulative proportion of hypertension (on the ordinate) compared to the cumulative proportion of the population categorized by socio-economic status (on the abscissa) [29]. When the curve coincides with the slope, it indicates that no socioeconomic-related inequality in hypertension. However, when the curve lies above the equality line, it signifies that hypertension is concentrated among the poor, thus exhibiting a pro-poor distribution. Conversely, if the curve falls below the equality line, it implies that hypertension is concentrated among the wealthy, indicating a pro-rich distribution [23,29].

### Decomposition analysis

To assess the relative contributions of each factor to inequalities in hypertension, we decomposed the concentration index of hypertension into its contributory factors using the Wagstaff approach [30]. In this decomposition, the elasticity of each determinant was estimated from a probit regression model, while its concentration index captured the distribution across socioeconomic groups. While O’Donnell recommend distinguishing between ‘need’ and ‘non-need’ factors to assess horizontal inequity in health [31], our study focused on measuring and decomposing overall socioeconomic inequality in hypertension. Therefore, we applied the Wagstaff-type decomposition without separating variables into need and non-need categories.

### Ethical consideration

DHS data is available to the public by request in different formats from the measure DHS website http://www.measuredhs.com. To conduct our study, we registered and requested the dataset from the DHS online archive and received approval to access and download the data files.

## Results

### Descriptive results

This study included a total weighted sample of 52,076 women of reproductive age with a mean age of 28.87 years with SD of 9.51. A total of 9,775 (18.77%) reproductive-age women had no formal education, and 36,270 (69.65%) were employed (Table 2).

**Table 2:** Characteristics of the study population with hypertension among reproductive-age women in five Sub-Saharan African countries.

Variables	Categories	Hypertension		
		Yes (%) n = 4,581 (8.80)	No (%) n = 47,494 (91.20)	Total weighted frequency (%)
**Age of women**	15-24	758 (3.85)	18,922 (96.15)	19,680 (37.79)
	25-34	1,469 (8.71)	15,403 (91.29)	16,873 (32.40
	35-49	2,353 (15.16)	13,168 (84.84)	15,522 (22.86)
**Marital status**	Married	4,025 (11.18)	31,994 (88.82)	36,020 (69.17)
	Not married	555 (3.46)	15,500(96.54)	16,055 (30.83)
**Occupation**	Working	768 (10.51)	32,457(89.49)	36,270 (69.65)
	Not working	768 (3.46)	15,037 (96.54)	15,805 (30.35)
**Women education status**	No education	710 (7.26)	9,065 (92.74)	9,775 (18.77)
	Primary	1,471 (9.98)	13,278 (90.02)	14,750(28.32)
	Secondary	1,855 (8.42)	20,164 (91.58)	22,019 (42.28)
	Higher	544(9.85)	4,985 (90.15)	5,529 (10.62)
**Sex of household head**	Male	2,977 (8.54)	31,869 (91.46)	34,846 (66.91)
	Female	1,604 (9.31)	15,625 (90.69)	17,229 (33.09)
**Wealth index**	Poorest	392 (4.69)	7,976 (95.31)	8,368 (16.07)
	Poorer	635 (6.78)	8,737 (93.22)	9,372 (18.00)
	Middle	852 (8.50)	9,172 (91.50)	10,024 (19.25)
	Richer	1,140 (9.91)	10,359 (90.09)	11,499 (22.08)
	Richest	1,561 (12.19)	11,248 (87.81)	12,810 (24.60)
**Mass media exposure**	No	820 (11.18)	6,521 (88.82)	7,342 (14.10)
	Yes	3,760 (8.41)	40,967 (91.59)	44,728 (85.90)
**Residence**	Urban	2,418 (9.87)	22,070(90.13)	24,489 (47.03)
	Rural	2,163 (7.84)	25,423 (92.16)	27,586 (52.97)

### Socioeconomic-related inequality in hypertension

**Fig 1:**
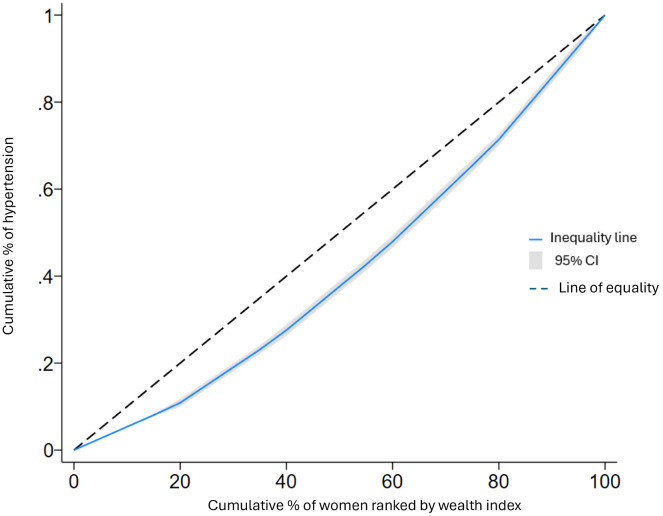
Concentration curve of hypertension among women of reproductive age in five SSA countries: DHS.

The concentration index (CI) for the pooled sample of five Sub-Saharan African countries was 0.057 (95% CI:0.049; 0.065). indicating a pro-rich inequality in hypertension among reproductive-age women. When disaggregated by country, the CI values were as follows: Benin: 0.072, Cameroon: 0.039, Ghana: 0.067, Kenya: 0.048, and Lesotho: 0.078. These results reveal that while all five countries exhibit pro-rich inequality in hypertension, the degree of inequality varies, with Lesotho and Benin displaying the highest levels (See S1 and S2 Figs). The concentration curve in Fig 1 consistently lies below the equality line, indicating that hypertension is more concentrated in households with a higher wealth index. Our results indicate significant socioeconomic inequalities in hypertension, with wealth quantile being the largest contributor. Unexpectedly, media exposure also emerged as a significant factor, suggesting the need for targeted health communication strategies.

### Decomposing the socioeconomic-related inequality in hypertension

According to the results of the decomposition, place of residence (Urban = ‒31.7%), marital status (married = ‒34.3%), media exposure (exposed for media = 18.9%), and total wealth index (125.9%), and richest (118.1%) were the main contributors to the pro-rich socioeconomic inequalities in hypertension among reproductive-age women (**Table 3**). The social determinants included in our model explained 83% of the estimated socioeconomic inequality in hypertension (**Table 3**).

**Table 3:** Contributing factors of socio-economic inequality of hypertension among reproductive-aged women in five Sub-Saharan Africa.

Variables		Coeff^*^	Elasticity	CI^**^	Absolute contribution	Percent Contribution	Sum percent contribution
**Age** *(Ref = 15–24 years)*	25-34	0.0189	0.0245	0.04	0.001	1.91	0.49
	35-49	0.0684	0.0815	‒0.0104	‒0.0008	‒1.47	
Subtotal					0.0003	0.49	
**Residence** *(Ref = Rural)*	Urban	-0.013	-0.025	0.7139	‒0.0183	‒**31.7**	-31.21
**Marital status** *(Ref = unmarried)*	Married	0.0619	0.1714	‒0.115	‒0.0198	‒**34.3**	-65.33
**Occupation** *(Ref = Not working)*	Working	0.0271	0.0756	‒0.0033	‒0.0002	‒0.4	-65.98
**Educational level** *(Ref = No Education)*	Primary	0.0217	0.0077	‒0.0579	‒0.0004	‒0.77	-63.64
	Secondary	0.0039	0.0066	0.2745	0.0018	3.17	
	Higher	-0.0063	-0.0027	0.2269	‒0.0006	‒1.06	
**Subtotal**					0.0008	2.33	
**Household head** *(Ref = Male)*	Female	0.0126	0.0167	0.0610	0.0010	1.77	-61.87
**Media exposure** *(Ref = No)*	Yes	-0.0143	-0.0491	‒0.2227	0.0109	**18.97**	-42.90
**Wealth index** (Ref = Poorest)	Poorer	0.0288	0.0207	‒0.35896	‒0.0074	‒12.89	**83.0354**
	Middle	0.0493	0.0380	‒0.0971	‒0.0036	‒6.39	
	Richer	0.0698	0.0617	0.2536	0.0156	27.11	
	Richest	0.0934	0.0919	0.7419	0.0682	118.11	
Subtotal		0.0728	66.18		**0.0728**	**125.94**	
Residuals		0.0625	56.82				
Total		0.11	100				

* Coefficient;

** Concentration index

## Discussion

This study presents comprehensive evidence on the socioeconomic inequality in hypertension among women of reproductive age in SSA. In addition, we explored the contributors to the observed socioeconomic inequality using decomposition analysis. The findings will provide valuable insights into the presence and magnitude of socioeconomic disparities in hypertension, enabling the development of evidence-based interventions and policies to address this inequality and improve health outcomes for women in SSA. Our findings align with previous studies indicating that wealth and education are major determinants of health inequalities. The significant role of media exposure also suggests that access to health information may mitigate these inequalities.

The concentration index of hypertension in SSA countries indicates a pro-rich inequality, as hypertensive individuals were more concentrated in higher socioeconomic status (SES) groups. This finding is in line with previous studies [12–15,17,32], consistently showing that the highest wealth quintiles were associated with a high likelihood of hypertension. This pro-rich inequality can be explained; individuals in higher wealth quantiles are more likely to exhibit obesity and overweight, which are known risk factors for hypertension [33,34]. Furthermore, the influence of wealth on healthcare access and utilization cannot be ignored. Individuals from higher wealth quantiles are more likely to have easier access to healthcare facilities, for screening, diagnosis, and treatment of hypertension, which contributes to a higher concentration of hypertension. conversely, the barrier to health care access in lower wealth quantiles results in underdiagnosis and lower concentration of hypertension within this population.

In contrast to our finding, several earlier studies reported that hypertension had pro-poor inequality [16–18,35,36]. These studies concluded that patients with lower wealth quantiles had higher concentrations of hypertension. These conflicting findings highlight the complexity of the relationship between wealth quantiles and the distribution of hypertension.

In this study, it was found that marital status played a significant role in contributing to socioeconomic inequality in hypertension. Approximately one-third of the observed hypertension inequality could be attributed to women’s marital status. This finding was consistent with other studies [37,38]. This could be attributed that marriage often brings about changes in lifestyle and dietary patterns change, the presence of marital responsibilities, which in turn lead to increased psychological and emotional stress, further impacting blood pressure levels. It is also worth considering that married women may visit health facilities more frequently for different medical conditions, which will lead to a higher detection rate of hypertension.

This study found that place of residence was another contributor to hypertension inequality in SSA countries. Urban residents had prominent levels of hypertension concentrations compared to rural residents. This was consistent with some previous studies [39–41]. The possible explanation for this finding is that women who reside in rural areas may lack information and basic knowledge about the risks of hypertension, limited access of health facilities, and low rates of screening for hypertension. This in turn leads to underreported hypertension and a lower concentration of hypertension in rural women compared to urban residents. Furthermore, dietary patterns in cities may be characterized by a larger consumption of processed and unhealthy foods, which are frequently heavy in sodium and saturated fats. These dietary patterns may contribute to the development of hypertension.

There were also conflicting reports on urban/rural disparities of hypertension, which hypertension had pro-poor distribution [42–44]. These studies indicated that the distribution of hypertension was more consistent with rural resident women. Therefore, conflicting findings highlight the need for further research in this area.

One of the strengths of this study is the use of data collected on the standardized data collection tools across countries which ensure the reliability of the findings. The second strength of this study is its broad geographic coverage, allowing for the generalizability of the findings to countries with similar settings. However, the study had some limitations. Limitations include potential biases in self-reported hypertension and the cross-sectional nature of DHS data, which precludes causal inferences. Additionally, our findings may not be generalizable to other SSA countries not included in this study. Furthermore, the other limitation of this study is that horizontal inequity was not explicitly estimated. While wealth-related disparities were observed and described using the terms ‘pro-rich’ and ‘pro-poor,’ this analysis did not formally assess horizontal inequity. Future studies could benefit from directly addressing this aspect to gain a more detailed understanding of how wealth influences healthcare access and outcomes across different wealth groups.

### Policy implications and future research

Policymakers should prioritize interventions that address socioeconomic determinants of health, such as improving access to education and health information. Tailored health communication strategies could also help reduce hypertension inequalities. Future research should explore longitudinal data to assess causal relationships between socioeconomic factors and hypertension. Studies focusing on other SSA countries and different age groups are also needed to generalize our findings.

## Conclusions

This study reveals significant socioeconomic inequalities in hypertension among reproductive-aged women in SSA, driven primarily by wealth and media exposure. The key contributors to this inequality were having a rich wealth index, being married, and urban residence. Targeted intervention should be implemented to reduce this socioeconomic inequality of hypertension.

## Supporting information

S1 Fig Concentration curves of hypertension among women of reproductive age (15–49 years) in Benin (A), Cameroon (B), and Ghana (C). (PDF)

S2 Fig Concentration curves of hypertension among women of reproductive age (15–49 years) in Kenya (D) and Lesotho (E). (PDF)
