# Supplementary material for: Socioeconomic inequality for hypertension among reproductive age women aged 15–49 from five Sub-Saharan Africa countries: A decomposition analysis of DHS Data
Source: PLOS Glob Public Health. 2025 Jul 2;5(7):e0004738. doi: 10.1371/journal.pgph.0004738 (PMC12221035; doi:10.1371/journal.pgph.0004738)
Supplement: S1 Fig — (PDF) [file pgph.0004738.s001.pdf]

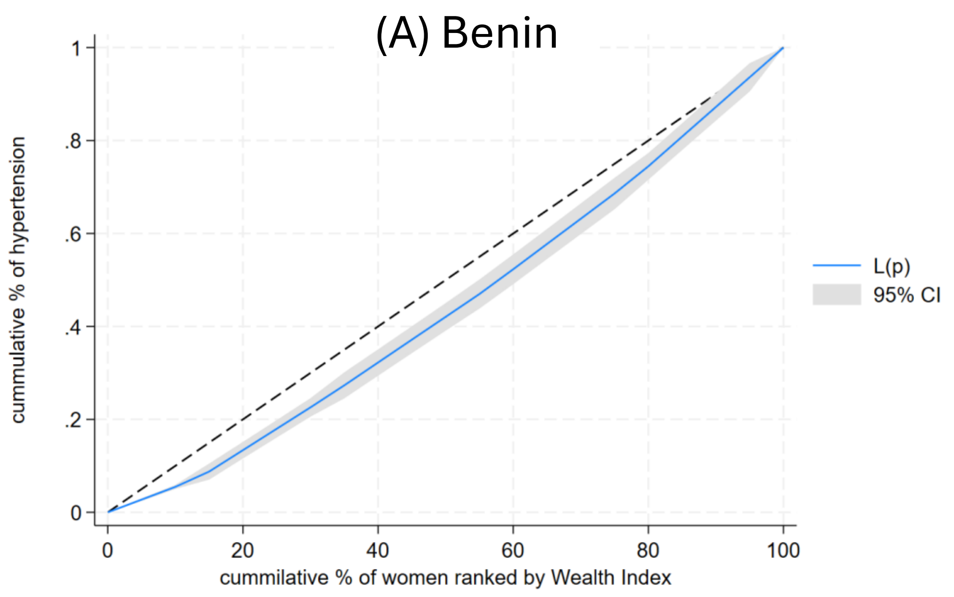

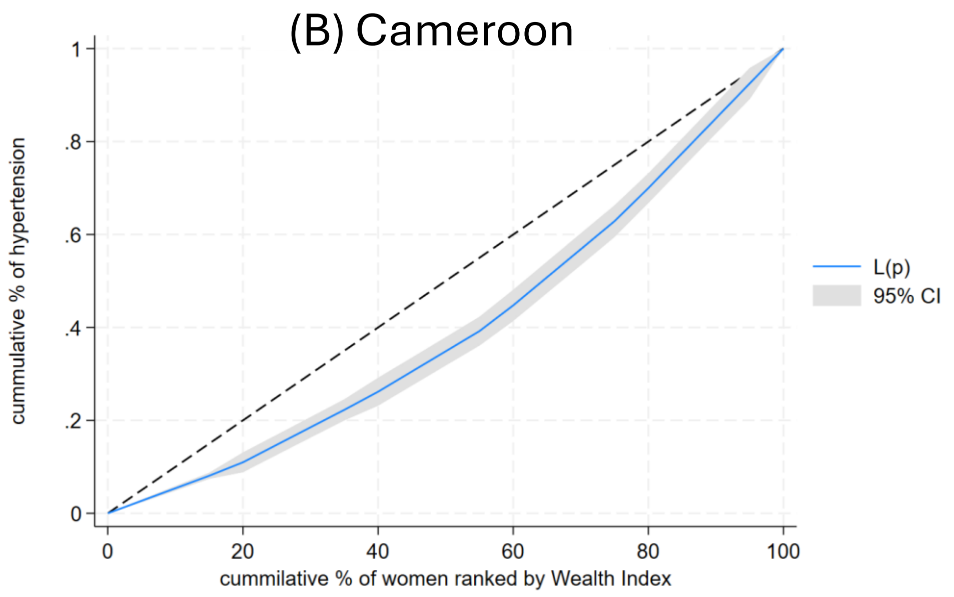

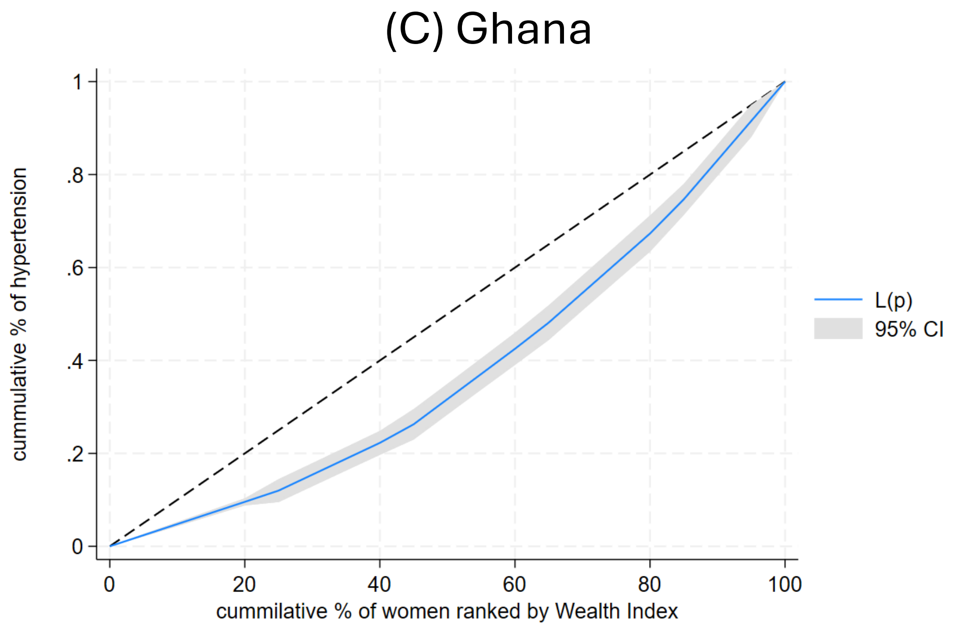

Supplementary Figure S1: Concentration curves of hypertension among women of reproductive age (15–49 years) in Benin (A), Cameroon (B), and Ghana (C).
