# Supplementary material for: Socioeconomic inequality for hypertension among reproductive age women aged 15–49 from five Sub-Saharan Africa countries: A decomposition analysis of DHS Data
Source: PLOS Glob Public Health. 2025 Jul 2;5(7):e0004738. doi: 10.1371/journal.pgph.0004738 (PMC12221035; doi:10.1371/journal.pgph.0004738)
Supplement: S2 Fig — (PDF) [file pgph.0004738.s002.pdf]

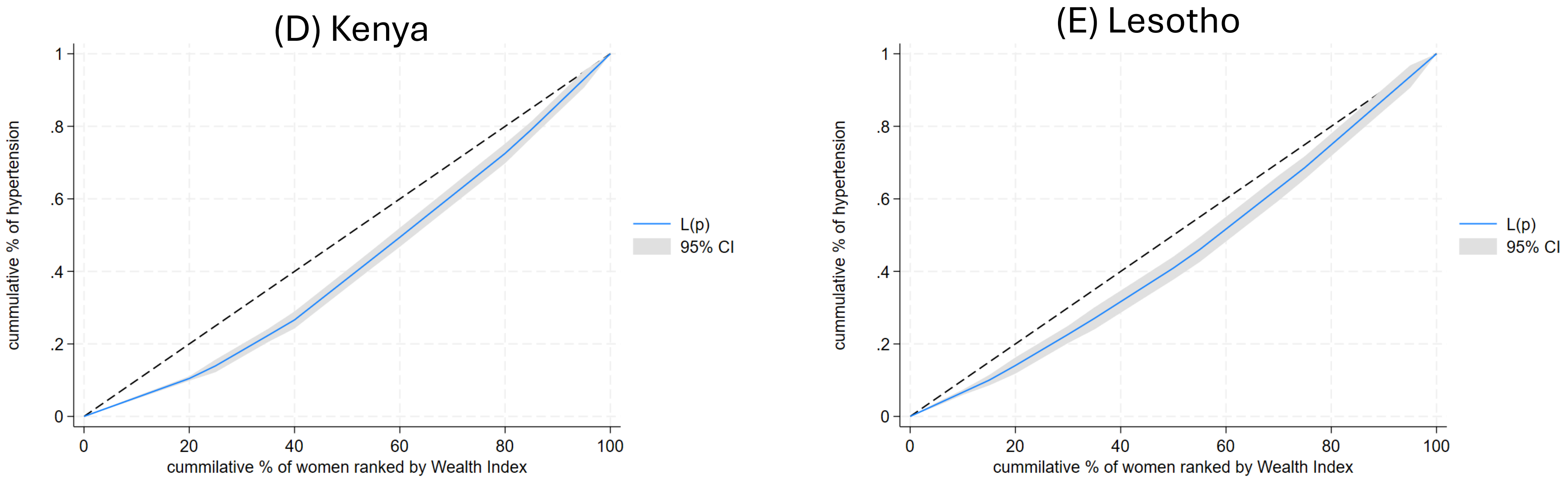

Supplementary Figure S2: Concentration curves of hypertension among women of reproductive age (15–49 years) in Kenya (D) and Lesotho (E).
